# 10.K. Workshop: School health during the COVID-19 Pandemic. Perspectives from the COVID-HL school principal study

**DOI:** 10.1093/eurpub/ckac129.655

**Published:** 2022-10-25

**Authors:** Organised by: EUPHA Working Group on Health Literacy, COVID-Health Literacy Network, Fulda University of Applied Sciences, Technical University Munich (Germany), Chair persons: Kevin Dadaczynski (Germany), Orkan Okan (EUPHA-HP)

## Abstract

The COVID-19 pandemic is associated with far-reaching challenges for the educational system, which impact the health of all involved in the school context, making it a critical public health topic. Consequences include school-closures, a switch from face-to-face classroom learning to homeschooling via online learning, resulting in uncertainty, stress and an increased risk of reinforcing already existing inequalities. Since the pandemic started, some research has been conducted in child and adolescent populations. However, there is very limited research available exploring the effects of the pandemic on school principals, who are responsible for all aspects of the school life and therefore have to cope with particularly high demands as a result of the COVID-19 pandemic. Pre-pandemic research shows that school principals report high work-related stress and more frequently psychological and physical burden compared to other professional groups (Dadaczynski et al., 2020; Phillips et al., 2008). Moreover, little attention has been paid to what health promotion activities are implemented by schools during pandemic times and what the schools’ needs are in this area. To provide empirical evidence on that matter, a school principal survey was conducted within the international COVID-Health Literacy Research Network (www.covid-hl.org), that aimed to assess (1) work-related stress and strain, (2) corona-specific health literacy among school principals and (3) the implementation status of activities in school health promotion during the COVID-19 pandemic. This workshop aims to present and discuss findings of the COVID-HL school principal study from Germany, Italy, Poland, Switzerland and Wales, which have used the same instrument and study design. The first presentation will focus on the pandemic as a potential disruptive event impeding the implementation of holistic activities on school health promotion and prevention. In their presentation, Chiara et al. analyse beliefs about vaccines among principals and its associations with COVID-19 information satisfaction. The third presentation originates from Poland and will introduce findings regarding work-related coping behavior and its association with mental health of school principals. While the fourth presentation from Switzerland focus on the relationship between health literacy and health promoting activities implemented by schools, Marchant et al. explores the effects of the COVID-19 pandemic on senior leaders experiencing high job demands. Each project will be given ten minutes to present their findings, including questions, which will be followed by Q&A and an open discussion with the audiences. This workshop offers a forum for researchers, practitioners and policy-makers interested in school health promotion and school staff health. By dialogue and two-way communication, vivid interaction will be ensured, allow building synergies, and facilitate networking and capacity building.

**Key messages:**

• School leaders should be systematically supported as ‘gate-keepers’ of school health promotion.

• School principals represent a largely neglected target group for school health promotion.

